# Electrohydrodynamic Coating with Acyclovir PLGA Conjugate for Antiviral Functionalization of Medical Surfaces

**DOI:** 10.3390/ijms262210983

**Published:** 2025-11-13

**Authors:** Tomasz Urbaniak, Witold Musiał

**Affiliations:** Department of Physical Chemistry and Biophysics, Pharmaceutical Faculty, Wroclaw Medical University, Borowska 211, 50-556 Wrocław, Poland; tomasz.urbaniak@umw.edu.pl

**Keywords:** drug-polymer conjugates, electrospinning, electrospraying, coating, sustained drug delivery, acyclovir

## Abstract

Sexually transmitted infections, notably herpes simplex virus, remain significant global health concerns. Localized delivery systems that provide sustained antiviral activity at mucosal surfaces offer an attractive alternative to systemic therapies. In this study, we developed electrohydrodynamically deposited coatings utilizing a covalent acyclovir–poly (lactic-co-glycolic acid) (ACV–PLGA) conjugate for potential antiviral functionalization of medical devices. The ACV–PLGA prodrug was synthesized via drug-initiated ring-opening polymerization, yielding a copolymer characterized by FTIR, NMR, GPC, and DSC, with controlled drug loading and biodegradable properties. Systematic optimization of electrospinning and electrospraying parameters enabled the fabrication of both particulate and nanofibrous coatings on silicone ring models. Morphological analysis by SEM demonstrated that polymer concentration, solvent composition, and applied voltage critically governed coating architecture, ranging from microparticle layers to uniform bead-free fibers. In vitro studies revealed morphology-dependent degradation profiles and sustained release of ACV over 56 days. This integrated approach combining covalent prodrug synthesis with tunable electrohydrodynamic deposition offers a promising strategy for long-acting local antiviral prophylaxis via functionalized medical surfaces.

## 1. Introduction

Sexually transmitted infections (STIs) continue to pose a major global health challenge, with herpes simplex virus (HSV) and human immunodeficiency virus (HIV) among the most prevalent viral pathogens [1,2]. Despite improvements in diagnostics and therapeutics, effective prevention remains critical for reducing transmission and disease burden. Long-acting, localized delivery systems have emerged as promising strategies, providing discreet, sustained protection at mucosal sites. The intravaginal ring exemplifies this approach; the dapivirine vaginal ring, designed for monthly HIV pre-exposure prophylaxis, demonstrated up to a 30% reduction in HIV acquisition in clinical trials, with higher efficacy linked to consistent use [3,4]. These results highlight the potential of intravaginal devices to maintain protective drug levels locally and overcome challenges associated with systemic treatments.

Extending such technologies to prevent other STIs, particularly HSV-2, is a logical advancement. HSV-2 infects an estimated 10–20% of adults worldwide, leading to recurrent lesions, psychological distress, and increased susceptibility to HIV [2]. Current management relies on oral antivirals like acyclovir (ACV), a guanosine analog that selectively inhibits viral DNA polymerase after phosphorylation in infected cells. While effective and well-tolerated, ACV’s clinical utility is limited by poor aqueous solubility, low oral bioavailability (~10–20%), and a short plasma half-life (~3 h), necessitating frequent dosing to maintain protective levels [5]. These pharmacokinetic drawbacks, along with documented resistance in immunocompromised patients, underscore the need for localized delivery systems that can sustain required mucosal drug concentrations [6].

Intravaginal formulations capable of prolonged ACV release could address these limitations by bypassing systemic circulation, reducing side effects, and maintaining steady drug levels at the site of infection risk [7]. In this context, long-acting intravaginal devices incorporating antiviral agents are an attractive alternative. Several intravaginal drug delivery systems have been developed to provide sustained local release of antiviral agents, particularly ACV and related compounds. Polymeric IVRs composed of silicone or ethylene–vinyl acetate matrices have been widely studied. Pod-IVRs containing ACV and tenofovir released ACV over 28 days in animal models [8]. Similarly, 3D-printed EVA rings loaded with 10–20 wt% ACV demonstrated an initial release burst in the first 24 h, followed by sustained drug release for up to 80 days [9]. Mucoadhesive polymer tablets and multilayer films using carrageenan, ethyl cellulose, chitosan, or xhantan gum have also been developed to prolong ACV residence time in the vaginal mucosa, achieving controlled release over several days [10,11]. These systems rely primarily on physical dispersion of ACV in non-degradable or swellable polymer matrices, and their release kinetics are governed by matrix hydration, swelling, and diffusion.

Electrohydrodynamic techniques, such as electrospinning and electrospraying, offer versatile methods for creating nanostructured coatings on biomedical devices [12]. These processes use high-voltage electric fields to produce either continuous fibers (electrospinning) or micro-/nanoparticles (electrospraying) from polymer solutions, enabling precise control over coating architecture, drug distribution, and release profiles. Their scalability, mild processing conditions, and compatibility with sensitive pharmaceuticals make them well suited for fabricating drug-eluting coatings on medical devices [13]. Electrospun nanofiber meshes provide high surface areas, tunable porosity, and have been applied to wound dressings, stents, and intravaginal rings to deliver drugs in a controlled manner [14]. Electrospraying, by contrast, yields uniform particle-based coatings that conform closely to complex surfaces, efficiently incorporating active agents [13]. Both methods are highly compatible with biodegradable polymers like poly(lactic-co-glycolic acid) (PLGA), supporting the fabrication of coatings that degrade while releasing therapeutic payloads [15].

Beyond physically loaded systems, covalent drug–polymer conjugates represent an advanced strategy for achieving predictable, site-specific, and sustained drug delivery. In these systems, the therapeutic molecule is chemically tethered to a polymer backbone through cleavable linkers, which prevents burst release, enhances stability, and ensures uniform drug distribution within the matrix [16]. Release is governed by polymer degradation or linker cleavage, offering more controlled kinetics than passive diffusion.

PLGA is particularly well suited for forming such conjugates due to its established biocompatibility, adjustable degradation rates, and straightforward synthesis via ring-opening polymerization (ROP) [15]. A notable strategy is drug-initiated ROP, wherein a drug bearing a nucleophilic group (such as a hydroxyl) serves directly as the initiator for polymerization of cyclic esters like lactide or glycolide. This approach simplifies synthesis, avoids post-polymerization modification steps, and provides precise control over drug loading determined by the monomer-to-drug feed ratio [17]. This strategy has been successfully applied to incorporate various hydroxyl-containing therapeutics, including paclitaxel, camptothecin, and ACV, into polyester chains. For example, ACV-initiated ROP of ε-caprolactone has produced poly-ε-caprolactone (PCL) conjugate-based micelles which exhibited a sustained release profile for 48 h [18]. Such systems illustrate the potential to create high-payload, covalently linked prodrugs that release their active agents through gradual hydrolysis, minimizing the burst effects common in physically encapsulated formulations [16].

The aim of this study was to develop a polymer–drug conjugate platform for antiviral medical device coatings. We synthesized an ACV–PLGA conjugate via drug-initiated ring-opening polymerization to combine the antiviral activity of ACV with the biodegradability of PLGA. Conjugate was subsequently processed into nanostructured coatings on silicone ring models using electrospinning and electrospraying, with the goal of prototyping a coating capable of long-term local prophylaxis against viral infection. By integrating covalent drug–polymer chemistry with electrohydrodynamic fabrication, this work establishes a versatile strategy for creating multifunctional medical device coatings that provide sustained antiviral protection.

## 2. Results and Discussion

### 2.1. ACV-PLGA Synthesis and Characterization

**Figure 1:**
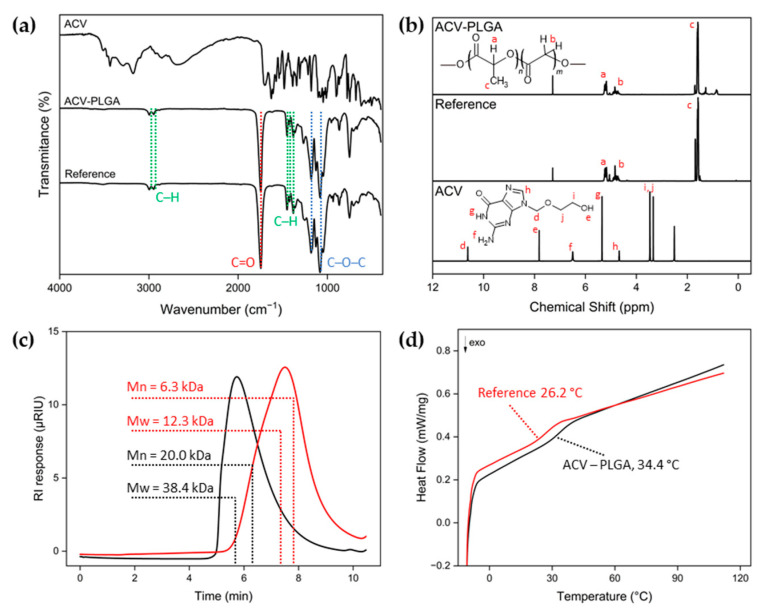
Physicochemical characterization of the ACV–PLGA conjugate and the reference polymer: (**a**) FTIR spectra of ACV, ACV–PLGA, and the reference synthesis product obtained without ACV; (**b**) ^1^H NMR spectra of ACV–PLGA and the reference polymer in CDCl_3_, with the ^1^H NMR spectrum of pure ACV recorded in DMSO-d_6_ shown for comparison. Letters indicate proton assignments: *a–c* correspond to PLGA backbone and methyl protons, while *d–j* denote ACV protons as labeled in the structure; (**c**) GPC chromatograms of ACV–PLGA (black) and the reference polymer (red); and (**d**) DSC thermograms of ACV–PLGA (black) and the reference polymer (red).

The use of active pharmaceutical ingredients and bioactive molecules as ROP initiators has been reported as a strategy to modify their pharmaceutical and pharmacokinetic properties. To function as an ROP initiator that triggers the growth of a polymer chain, the molecule must possess a hydroxyl group. Consequently, the resulting chain length depends directly on the ratio of monomers to initiator molecules. This ability to predetermine drug loading by stoichiometry is a noted benefit of drug-initiated ROP; for instance, Tong et al. demonstrated that varying the LA/drug ratio allows precise control over paclitaxel or camptothecin content in the resulting polymer–drug conjugates [17,19]. The resulting macromolecule, inherently loaded with the drug, retains properties similar to the pure polymer and can be processed using various techniques. The product obtained in ACV-initiated copolymerization of LA and GA was characterized by spectral and thermal methods (Figure 1).

While the high yield of 90% indicates efficient ACV-initiated polymerization, confirmation of covalent attachment remains challenging due to the very low theoretical drug content in the synthesized conjugate. The reference reaction, conducted under identical conditions but without acyclovir, resulted in a yield of 57.3%, suggesting that the presence of ACV affected the polymerization kinetics. Based on the monomer-to-initiator ratio, the theoretical loading was 0.225%, while accelerated degradation in 0.5 M NaOH confirmed an actual loading of 0.163% (Figure S1). The maximum ACV concentration during alkaline hydrolysis coincided with the complete dissolution of the polymer sample, confirming hydrolysis-driven release, while the control experiment with free ACV under identical conditions verified the drug’s stability in the degrading conditions. FTIR spectra of the ACV–PLGA conjugate and the reference synthesis product displayed identical profiles, dominated by the characteristic PLGA absorptions: a strong ester C = O stretching band at 1746 cm^−1^, C–O–C stretching at 1181 and 1083 cm^−1^, and CH/CH_2_ stretching at 2995 cm^−1^ and 2946 cm^−1^, together with bending bands at 1452 cm^−1^, 1424 cm^−1^ and 1383 cm^−1^ (Figure 1a) [20]. No distinct peaks attributable to ACV were observed, which is consistent with its low, determined experimental loading. At such concentrations, drug-related absorptions are fully masked by the much stronger polymer vibrations. This observation agrees with reports showing that ATR-FTIR typically detects minor organic components in polymer matrices only above ~0.3–1 wt % [21]. The ^1^H NMR spectrum of ACV–PLGA recorded in CDCl_3_ exhibited the typical broad resonances of the polymer backbone, with peaks at δ 1.59 ppm, 5.20 ppm, and 4.79 ppm corresponding to the methyl, methine, and methylene protons of the lactide and glycolide units, respectively (Figure 1b). No distinct peaks assignable to ACV were observed due to its extremely low molar fraction and overlap with broad polymer resonances. ^1^H NMR analysis of previously described PCL-based ACV conjugates (Mn ≈ 17 kDa) likewise showed poorly discernible drug-derived peaks embedded within the baseline [18]. The calculated LA:GA ratio of 80:20 closely matched the monomer feed composition, confirming controlled copolymerization. GPC analysis revealed Mn = 20.0 kDa and Mw = 38.4 kDa for ACV–PLGA, whereas the reference polymer showed markedly lower values (Mn = 6.3 kDa, Mw = 12.3 kDa) (Figure 1c). These results indicate that ACV acted as an efficient initiator, promoting higher conversion and longer chain formation compared with the spontaneous reference reaction. The observed molecular weight of ACV–PLGA was, however, considerably lower than the theoretical value predicted from the monomer-to-initiator feed ratio (~100 kDa), which can be attributed to competitive initiation by trace impurities and side reactions similar to those likely responsible for polymer formation in the reference synthesis and potential simultaneous initiation from both hydroxyl and amino groups on ACV, effectively producing a larger number of shorter polymer chains [22]. DSC analysis showed a Tg of 34.4 °C for the ACV–PLGA sample and 26.2 °C for the reference polymer, with the lower value of the latter most likely resulting from its lower molecular weight (Figure 1d) [23]. A Tg near physiological temperature suggests that the conjugate polymer may exhibit enhanced segmental mobility under in vivo conditions, which could influence both mechanical stability and drug-release behavior. Together, these findings confirm that ACV actively participated in the polymerization process and influenced the molecular characteristics of the resulting copolymer.

### 2.2. Electrohydrodynamic Processing of ACV-PLGA Prodrug

The synthesized ACV–PLGA conjugate had a relatively low molecular weight, placing it near the lower end of typical ranges reported for electrospinnable biodegradable polymers. Such molecular weights are well below values often preferred for robust fiber formation, as numerous studies have shown that higher molecular weights (e.g., PLGA ~100 kDa or PCL > 45 kDa) readily form uniform nanofibers at moderate concentrations due to abundant chain entanglements [24,25]. In contrast, lower Mw macromolecules demand higher solution concentrations or carefully chosen solvent systems to achieve sufficient overlap and viscoelasticity to suppress Rayleigh instabilities and enable stable jet elongation [26]. Therefore, electrohydrodynamic processing of the ACV–PLGA conjugate was systematically studied to elucidate how processing conditions shape the resulting morphologies. Across the experimental matrix, three principal morphologies were observed depending on the interplay of polymer concentration, solvent system, and applied voltage: (i) electrosprayed droplets, (ii) beaded fibers, and (iii) continuous, bead-free nanofibers (Figures S2–S7, Table 1).

**Figure 2:**
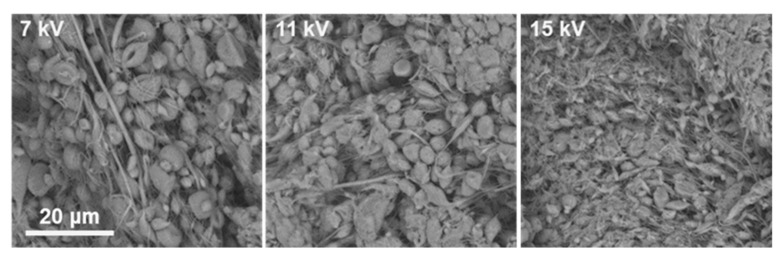
SEM images showing the voltage-dependent morphology of ES4 coatings obtained at 7 kV, 11 kV, and 15 kV, the scale bar is the same for all micrographs.

At 25% *w*/*v* ACV–PLGA, all solvent systems and voltages led predominantly to electrospraying or bead-on-string structures. For example, in DMF:CHCl_3_ (3:5), increasing voltage transitioned the morphology from dispersed particles mixed with sparse fibers to beaded, thin filaments without achieving stable fiber formation (Figure 2).

**Figure 3:**
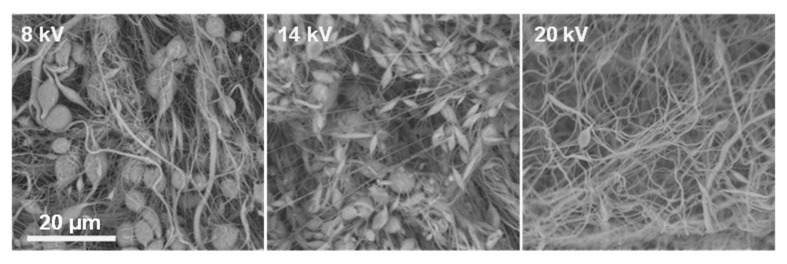
SEM images showing the voltage-dependent morphology of ES8 coatings obtained at 8 kV, 14 kV, and 20 kV, the scale bar is the same for all micrographs.

This is consistent with insufficient chain entanglement below the critical concentration necessary for electrospinning. Commercial PLGA solutions below the entanglement threshold failed to form fibers, instead producing particles or beaded morphologies, driven by surface tension overcoming viscous forces in the jet [24]. Increasing the polymer concentration to 30% *w*/*v* resulted in marked improvements in fiber formation, although uniform fibers were not consistently achieved. In DMF:THF systems (both 5:5 and 3:5), electrospinning typically yielded beaded fibers across voltages from 7 up to 19 kV. This suggests that the solution viscosity was approaching the level needed for stable jet elongation but still insufficient to completely suppress Rayleigh instabilities. In DMF:CHCl_3_ systems, however, the morphology changed notably. The 30% solution with DMF:CHCl_3_ (3:5) at the highest tested voltage produced the first instances of predominantly uniform, bead-free nanofibers, indicating that CHCl_3_-containing solvent enhanced chain extension and reduced the overlap concentration needed for entanglement (Figure 3).

At 35% *w*/*v*, the solutions generally achieved the entanglement threshold necessary for fiber formation across all solvent systems, but the quality of fibers depended on solvent composition. In DMF:THF (3:5), fibers were primarily beaded at lower voltages but transitioned to uniform nanofibers at higher voltages (18–19 kV), reflecting a voltage-driven enhancement of electrostatic stretching that stabilizes the Taylor cone and suppresses bead formation. DMF:CHCl_3_ (3:5) similarly yielded beaded fibers across 7–17 kV, but produced uniform fibers at 18 kV, reinforcing the role of electric field strength in overcoming surface tension-driven jet breakup.

**Figure 4:**
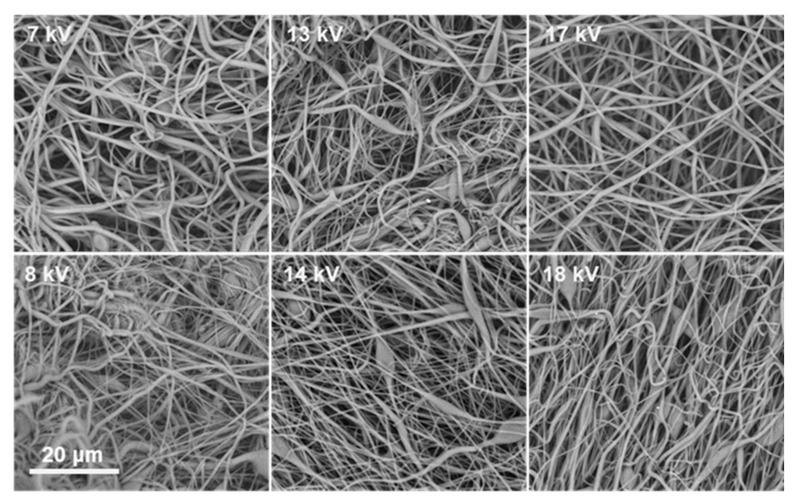
SEM images showing the voltage-dependent morphology of ES14 coatings obtained at 7 kV, 13 kV, 17 kV (**upper row**) and ES15 coating obtained at 8 kV, 14 kV, 18 kV (**bottom row**), the scale bar is the same for all micrographs.

At 40% *w*/*v* ACV–PLGA, nearly all tested solvent combinations facilitated formation of continuous fibers, with solvent choice primarily modulating defect frequency. Notably, DMF:THF (3:5) and DMF: CHCl_3_ (3:5) at 40% produced predominantly bead-free fibers even at moderate voltages (7–17 kV), underscoring that once the solution is above the entanglement concentration, process robustness increases significantly (Figure 4).

This parallels observations by Liu et al., where high-concentration PLGA solutions demonstrated wide operational voltage ranges yielding defect-free fibers, attributed to dominant viscoelastic forces suppressing capillary instabilities [20]. Across all conditions, increasing the proportion of CHCl_3_ relative to DMF consistently improved fiber uniformity at lower polymer concentrations, likely due to reduced surface tension and enhanced polymer coil expansion.

The optimization revealed that both polymer concentration and solvent selection are critical to achieving uniform nanofibers, with higher concentrations ensuring sufficient chain entanglements, while CHCl_3_-rich solvent systems reduce the minimum required concentration for smooth fiber formation. Voltage was also pivotal, with higher fields helping to stabilize the jet and eliminate bead defects. These systematic findings provided essential insights to guide subsequent fabrication of ACV–PLGA nanofiber coatings on silicone rings under these refined conditions.

### 2.3. Silicone Ring Coating

**Figure 5:**
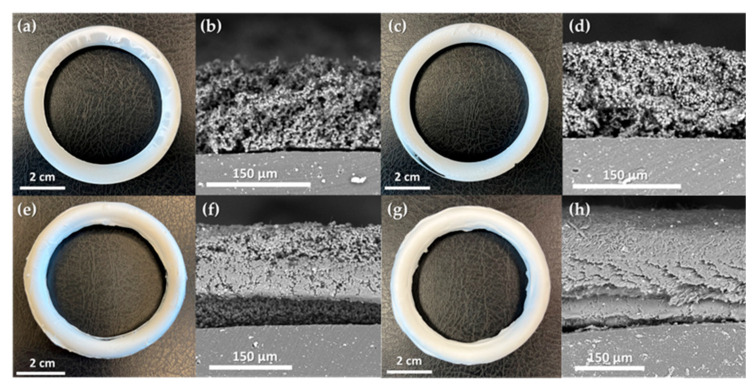
Macroscopic views and SEM cross-sections of coated silicone rings R1–R4: R1 (**a**,**b**), R2 (**c**,**d**), R3 (**e**,**f**), and R4 (**g**,**h**). For each ring, the left image shows the macroscopic appearance, and the right image shows the coating morphology.

Model silicone rings were coated with an ACV–PLGA prodrug using electrohydrodynamic deposition, applying parameters established through preceding screening. This process produced four distinct coating types: two particulate coatings (R1, R2) and two fibrous coatings (R3, R4). Specifically, R1 and R2 were prepared using the ES3 formulation at 10 kV and 16 kV, respectively, while R3 employed ES8 at 20 kV and R4 used ES14 at 15 kV, as defined in Table 1. Each coating achieved mostly continuous coverage of the toroidal substrate (Figure 5), demonstrating that even a simple static setup (fixed needle, non-rotating collector) can effectively coat 3D structures.

This underscores the potential of applying drug-loaded layers to complex devices post-fabrication, beyond incorporating drug solely into the core.

The particulate coatings (R1, R2) formed microsphere layers with different packing densities, thicknesses, and corresponding deposited masses. R1, produced at a lower voltage (10 kV), resulted in a relatively thick (~180 ± 38 μm), porous layer of loosely interconnected particles, with an associated mass of ~0.188 g per ring. In contrast, R2, obtained at a higher voltage (16 kV), was thinner (~116 ± 28 μm) yet comprised more compactly merged particles, and correspondingly held slightly less deposited prodrug (~0.167 g). The denser packing in R2 likely arose from the increased acceleration and impact of droplets under the stronger electric field. The fibrous coatings (R3, R4) displayed typical electrospun morphologies and supported more efficient polymer deposition. R3 formed a beaded nanofiber mesh (~102 ± 27 μm) with an overall mass of ~0.298 g. Interestingly, R4, despite being prepared under conditions that in planar trials produced smooth, bead-free fibers, exhibited elongated bead-like enlargements along otherwise uniform fibers, yielding a subtly irregular texture and the highest mass among the coatings (~0.308 g). This shift in morphology is likely due to the insulating effect of the accumulating polymer on the ring surface, locally altering the electric field and destabilizing the jet. Cross-sectional observations confirmed good adhesion of all coatings to the silicone substrate. However, the particulate layers (R1, R2) showed weak interparticle cohesion, separating cleanly and maintaining distinct boundaries. In contrast, the fibrous coatings (R3, R4) were strongly entangled, and in consequence, deformed during cutting rather than fractured, indicating robust internal connectivity. Tight fiber network suggests enhanced mechanical resilience, likely improving coating stability under physiological conditions.

### 2.4. Coating Degradation and ACV Release In Vitro

The degradation behavior of PLGA-based systems is well documented, with both microparticles and electrospun fibers known to undergo hydrolytic bulk erosion, leading to gradual loss of structural integrity, polymer molecular weight reduction, and release of incorporated drugs over weeks to months [27,28]. To evaluate how the different coating architectures influence these processes in the case of ACV-PLGA conjugate, we monitored the morphological evolution, molecular weight, and ACV release profiles of the R1–R4 coatings over a 56-day period. This combined analysis provides insights into how particulate versus fibrous morphologies modulate degradation and drug release kinetics, informing the design of durable, long-acting delivery systems.

#### 2.4.1. Hydrolytic Degradation of ACV-PLGA Coatings

**Figure 6:**
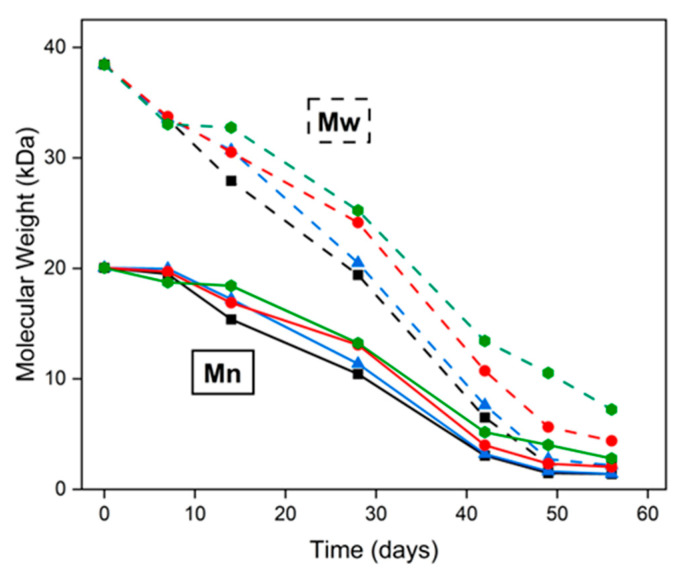
Time-dependent changes in Mn (solid lines) and Mw (dashed lines) of ACV-PLGA coatings with different morphologies: particulate R1 (black squares), R2 (blue triangles) and fibrous R3 (red circles), R4 (green hexagons) during in vitro incubation in PBS at 36.6 °C.

The coatings underwent a substantial decrease in molecular weight over the 8-week degradation period, indicative of hydrolytic backbone cleavage. Both Mn and Mw showed marked reductions, reflecting progressive chain scission. Mw dropped more steeply than Mn during the first month, which is consistent with random hydrolytic cleavage of longer polymer chains (Figure 6).

In initial PLGA degradation phase, the polymer chains are cleaved randomly along the backbone, causing a significant reduction in average molecular weight without immediate mass loss or generation of many small fragments [29]. By day 28, Mw had decreased by roughly 50–60% across all coatings, reaching ~22–25 kDa for particulate coatings (R1, R2) and ~28–30 kDa for fibrous coatings (R3, R4). The steeper decline in Mw compared to Mn suggests that the largest polymer chains were cleaved early in the degradation process. Correspondingly, we observed Mn around ~10–11 kDa for the particulate coatings (R1, R2) and ~13 kDa for the fibrous coatings (R3, R4) at 28 days. Notably, the particulate coatings (R1, R2) degraded faster than the fibrous ones (R3, R4). This is evidenced by the lower molecular weight of R1–R2 compared to R3–R4 at each time point (e.g., Mn ~10–11 kDa vs. ~13 kDa on day 28). The faster molecular weight reduction in R1–R2 is likely due to their higher surface area-to-volume ratio, which facilitates water ingress and polymer–water contact, accelerating hydrolytic cleavage throughout the matrix [15].

#### 2.4.2. Coating Morphology Changes During Degradation

**Figure 7:**
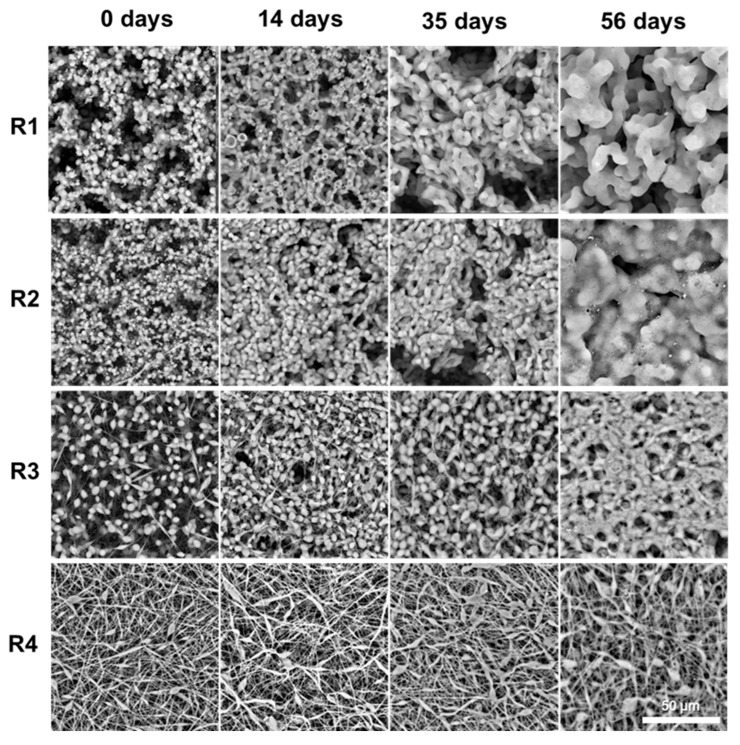
SEM images of R1–R4 ACV–PLGA coatings on silicone rings at successive time points during in vitro incubation in PBS at 36.6 °C, the scale bar is the same for all micrographs.

SEM imaging revealed consistent structural changes across all coating types, driven by polymer relaxation and merging during degradation (Figure 7).

In particulate coatings (R1, R2), initially discrete PLGA microspheres gradually coalesced into a smoother, interconnected network. In fibrous coatings (R3, R4), fibers thickened and fused at contact points, leading to partial loss of the fibrous network and a transition toward a more heterogenous morphology. These transformations coincided with a steady decrease in molecular weight, as confirmed by GPC. As chain scission progressed, the lowering of the polymers molecular weight led to a corresponding reduction in Tg. This plasticization effect made the polymer more susceptible to flow and deformation at 36.6 °C, facilitating particle merging and fiber fusion. The morphological evolution supports the release data: as the coatings soften and reorganize, surface accessibility and internal mobility increase, sustaining ACV release.

#### 2.4.3. Acyclovir In Vitro Release Profile

The release profiles of ACV from all four ACV–PLGA coatings (R1–R4) demonstrated extended drug release over 56 days, characteristic of a degradation-driven mechanism. Given the covalent linkage of ACV to the PLGA backbone, drug release is expected to occur primarily via hydrolysis of the polymer chain, rather than passive diffusion. This was supported by the absence of a sharp initial burst, particularly in the fibrous coatings.

**Figure 8:**
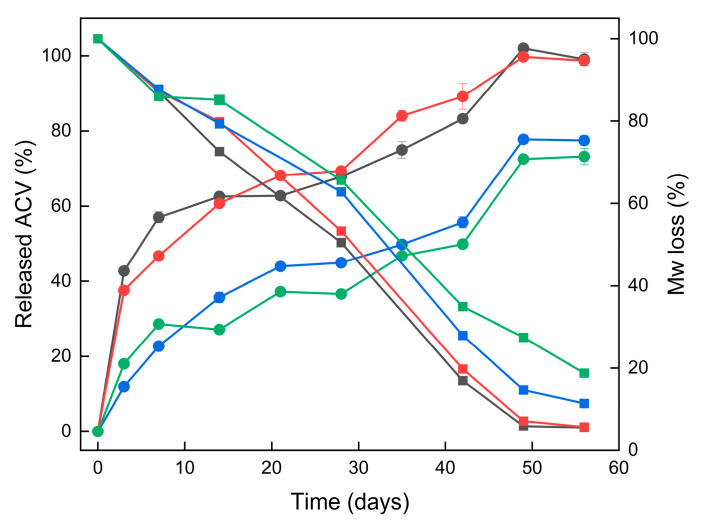
Comparative profiles of cumulative acyclovir release (circles, left y-axis) and polymer Mw loss (squares, right y-axis) from particulate R1 (black), R2 (red), and fibrous R3 (blue), R4 (green) ACV–PLGA coatings during 56 days of incubation in PBS (pH 7.4, 36 °C).

A closer look at the early time points (day 3) reveals that particulate coatings R1 and R2 released approximately 43% and 38% of their total released drug, respectively—indicating a moderate initial burst—while fibrous coatings R3 and R4, releasing only 12% and 18%, showed a more gradual onset of release (Figure 8).

The higher surface area likely contributed to the faster initial erosion and drug release. SEM imaging revealed early-stage softening and merging of both particle and fiber morphologies. GPC data confirmed that molecular weight degradation progressed more rapidly in R1–R2 compared to R3–R4, aligning with their earlier ACV release. Kinetic fitting of the release data to zero-order, first-order, Higuchi, and Korsmeyer–Peppas models supported predominantly diffusion- and erosion-controlled behavior (Table S1). First-order and Higuchi relationships provided the best overall agreement, although differences among models were minor, implying concurrent degradation and diffusional processes rather than a single dominant mechanism. Complex kinetic behavior observed for PLGA-based systems arises from the interplay of hydrolysis, autocatalysis, and water diffusion occurring on overlapping timescales, which makes the overall degradation appear as a superposition of several concurrent processes [20,30]. The observed kinetics contrast with physically loaded systems, where burst release is frequently reported. For example, Wang and Windbergs showed that physically encapsulated ACV in PLGA nanofibers led to significant release within 24 h unless coaxial structures or hydrophilic excipients were incorporated [31]. Similarly, Shen et al. described ACV-loaded PLGA nanofiber mats that released the full drug dose in under 21 days, with initial losses strongly influenced by matrix porosity and drug distribution [32]. In our system, despite the relatively low overall drug loading, sustained release was achieved across all morphologies. A key distinction of the covalent conjugate approach is the absence of formulation additives or multi-layer spinning processes to control release. Literature on other drug–PLGA conjugates (e.g., paclitaxel or camptothecin prodrugs) similarly reports predictable, erosion-controlled release [33]. The therapeutic relevance of the released ACV concentrations can be evaluated by comparison with reported antiviral susceptibility data. Studies of HSV-1 and HSV-2 clinical isolates show mean IC_50_ values around 0.02–2.2 µg/mL [34]. In a intravaginal ring study, cervicovaginal lavage concentrations of ACV reached approximately 0.4 µg/mL after 7–14 days of use—comparable to or exceeding levels achieved by oral valacyclovir, but with negligible systemic exposure [35]. The concentrations released from the described coatings therefore fall within the pharmacologically active range, supporting their potential to maintain local antiviral activity during prolonged degradation.

These findings support the use of conjugate-based electrohydrodynamic coatings as a promising platform for long-acting antiviral delivery, eliminating the need for complex formulation strategies while improving release predictability. While in vitro release tests revealed no coating exfoliation, apparent brittleness at room temperature highlights the need for adhesion and peeling assessments under physiologically relevant conditions. Further studies are also required to evaluate in vivo performance, biocompatibility, and antiviral efficacy, but the use of electrohydrodynamic methods for coating deposition provides a flexible approach for integrating such systems into drug-eluting devices designed for mucosal delivery.

## 3. Materials and Methods

### 3.1. Materials

L-lactide (LA, 95%) and glycolide (GA, 97%) were purchased from BLDpharm (Shanghai, China), recrystallized from ethyl acetate, and stored in a desiccator prior to use. ACV was kindly provided by HASCO-Lek (Wrocław, Poland) and dried using a moisture analyzer before use. Dimethylformamide (DMF), chloroform, tetrahydrofuran (THF), and triethylamine were obtained from Chempur (Piekary Śląskie, Poland). Tin(II) 2-ethylhexanoate was purchased from Sigma-Aldrich (St. Louis, MO, USA). Deuterated chloroform and deuterated dimethyl sulfoxide were obtained from Deutero (Kastellaun, Germany). Phosphate-buffered saline was obtained from Pol-Aura (Morąg, Poland). GPC polystyrene calibration standards were purchased from Supelco (Bellefonte, PA, USA). All solvents and reagents were of analytical grade and used without further purification unless otherwise specified.

### 3.2. Synthesis of ACV-PLGA Prodrug

The ACV-PLGA covalent prodrug was synthesized via ROP of LA and GA using ACV as the initiator and stannous octoate (Sn(Oct)_2_) as the catalyst. Polymerization was performed in glass three-necked flasks sealed with septa under a constant nitrogen atmosphere. Specifically, 8.325 g of LA, 1.675 g of GA, and 0.225 g of ACV were weighed to achieve an LA:GA molar ratio of 75:25, targeting a number-average molecular weight of 100 kDa, based on the monomer-to-initiator ratio. Sn(Oct)_2_ (23.35 µL) was then added to reach a catalyst-to-monomer molar ratio of 1:1000. Reaction mixture was stirred and heated to 130 °C, after which polymerizations proceeded for 24 h under continuous nitrogen flow. The crude polymer was then dissolved in CHCl_3_ and precipitated dropwise into cold methanol. The resulting polymer was collected by vacuum filtration and dried under reduced pressure at room temperature for 48 h. A reference reaction was conducted under identical conditions as the ACV–PLGA synthesis but without the addition of ACV, yielding a blank PLGA sample used as a control in subsequent analyses.

### 3.3. Fourier-Transform Infrared Spectroscopy

FTIR spectra were acquired at room temperature using a Thermo Scientific Nicolet iS50 spectrometer (Thermo Fisher Scientific, Waltham, MA, USA) equipped with a diamond ATR accessory. Spectra were recorded in the range 4000–400 cm^−1^ at a resolution of 4 cm^−1^, averaging 32 scans per sample. Samples were measured directly on the ATR crystal without additional preparation. Baseline correction was performed using the OMNIC™ software (version 9.3.30), and spectra are presented in transmittance mode.

### 3.4. ^1^H Nuclear Magnetic Resonance Spectroscopy

^1^H NMR spectra were recorded on a Bruker AVANCE III™ 600 MHz spectrometer at 298 K. Samples (~10 mg) were dissolved in 0.7 mL of CDCl_3_ (for ACV–PLGA) or DMSO-d_6_ (for ACV). Spectra were acquired using a zg30 pulse sequence with a 30° flip angle, a relaxation delay of 2 s, and 16 scans per sample. Chemical shifts (δ) are reported in ppm and referenced to the residual solvent signals (7.26 ppm for CHCl_3_, 2.50 ppm for DMSO). Monomer composition was determined by integration of characteristic peaks.

### 3.5. Differential Scanning Calorimetry

Thermal transitions were analyzed using a DSC 214 Polyma instrument (Netzsch, Selb, Germany). Approximately 5 mg of synthesized conjugate was sealed in an aluminum pan with a pierced lid. Sample was scanned from −10 °C to 120 °C at a heating rate of 5 °C/min under a nitrogen flow of 250 mL/min. Two heating/cooling cycles were performed, with the second heating conducted in modulated mode (±0.5 °C every 60 s). The glass transition temperature (Tg) was determined from the inflection point of the heat flow curve in the second heating run using Proteus software (version 7.1.0) (Netzsch, Selb, Germany).

### 3.6. Gel Permeation Chromatography

Number-average molecular weights (Mn), weight-average molecular weights (Mw), and dispersity indices (PDI) were determined using a Thermo Scientific Dionex UltiMate 3000 GPC system (Thermo Fisher Scientific, Waltham, MA, USA) equipped with a RefractoMax 521 RI detector (ERC Inc., Saitama, Japan). Samples (5 mg/mL in DMF) were injected (100 µL) onto a Phenogel column (10^3^ Å, 5 µm, 300 × 7.5 mm) at 25 °C, with THF as the eluent at a flow rate of 1.0 mL/min. Molecular weights were calculated relative to a calibration curve constructed from polystyrene standards ranging from 1050 to 96,000 g/mol.

### 3.7. Coating Fabrication by Electrospinning

Initial optimization of the electrohydrodynamic deposition process was performed using standard planar collector on E-Fiber EF050 electrospinning instrument (SKE, Bollate, Italy). For each deposition condition, small polymer samples were collected and examined by SEM to assess morphology. Details of the solvent systems, polymer concentrations, and voltages explored are provided in Table 1. Following optimization, silicone rings (54 mm in diameter and 8 mm cross-sectional thickness; Kramp, Varsseveld, The Netherlands) were coated under selected conditions. Prior to coating, rings were sequentially cleaned by immersion in acetone (3 × 10 min), methanol (10 min), and distilled water (10 min), each step performed in an ultrasonic bath. Rings were then suspended directly in front of the collector using a dedicated 3D-printed holder, ensuring stable positioning and contact. Each side was coated for 1 h, then rotated to achieve uniform coverage. The mass of the deposited coating was determined by measuring the weight difference between the uncoated ring and the fully coated ring after both sides were processed. The coating process for both the optimization experiments and the ring applications was performed under the same base conditions: a 10 cm needle-to-collector distance, a flow rate of 1 mL/h, and an R17 stainless steel needle.

### 3.8. Scanning Electron Microscopy

SEM was used to characterize the coating morphology, including surface features, cross-sections, and changes during degradation. Images were acquired with a Phenom ProX G6 SEM (Thermo Fisher Scientific, Waltham, MA, USA) equipped with a backscattered electron detector, operated in low-vacuum mode without sample sputter coating. For cross-sectional analysis, small fragments of coated rings were carefully cut with a scalpel, and coating thickness was measured directly from SEM images at a minimum of five different points per sample. An accelerating voltage of 10 kV was used for initial process screening, while 15 kV was applied for imaging degradation samples and cross-sections.

### 3.9. In Vitro Degradation and Drug Release Analysis

An accelerated degradation study was performed to confirm the presence of covalently bound ACV in the ACV–PLGA conjugate, estimate the final drug loading, and assess the stability of ACV under alkaline conditions. Conjugate sample (100 mg) was incubated in 10 mL of 0.5 M NaOH at room temperature under continuous agitation. At predetermined time intervals, aliquots of the supernatant were collected, neutralized, and analyzed by HPLC to determine the cumulative amount of released acyclovir. In parallel, a control solution containing free acyclovir (0.200 mg/mL) was subjected to identical conditions to verify the absence of drug degradation in the basic medium.

Coated silicone rings were sectioned to enable parallel assessment of drug release, polymer degradation, and morphological changes. For drug release experiments, three-quarters of each ring were placed in screw-capped glass vials containing 10 mL of phosphate-buffered saline (PBS, pH 7.4). Vials were sealed with Parafilm and incubated in a water bath at 36.6 °C. At predetermined time points, 0.2 mL samples of the incubation medium were withdrawn and replaced with fresh PBS. The concentration of released ACV was determined by HPLC using a HITACHI Primaide system equipped with a UV-Vis detector set at 255 nm. Chromatographic separation was performed on a LiChrospher 100 RP8 column (250 × 4.6 mm I.D., 5 µm particle size) with a mobile phase consisting of 0.1% (*v*/*v*) triethylamine in water (pH 2.5, adjusted with orthophosphoric acid). The injection volume was 10 µL. For each coated ring, drug concentrations at each time point were determined by three independent HPLC injections, and data are reported as mean ± SD to represent measurement variability. For morphological evaluation, the remaining one-quarter of each ring was incubated in 20 mL PBS under identical conditions. At each time point, a piece was removed, rinsed, dried, and examined by SEM to assess degradation-induced structural changes. For molecular weight analysis, approximately 12 mg of coating fragments were incubated in 0.5 mL PBS under the same conditions. At selected time points, samples were collected, rinsed with distilled water, dried, and analyzed by GPC to monitor changes in polymer molecular weight.

Drug release data were fitted to linearized kinetic models by least-squares regression with a free intercept to account for the initial burst phase. The applied models included zero-order, first-order, Higuchi, and Korsmeyer–Peppas, with the corresponding linear equations presented in Table S1 [36]. From each regression, the rate constant (K or n) and the coefficient of determination (R^2^) were calculated to assess the goodness of fit. The Korsmeyer–Peppas model was evaluated within the early release region (M_t_/M_∞_ ≤ 0.6) to ensure the validity of the diffusion exponent n.

## 4. Conclusions

In this study, a covalent ACV–PLGA conjugate was synthesized via drug-initiated ring-opening polymerization using a 75:25 LA:GA monomer feed, yielding a polymer with Mn ≈ 20.0 kDa, Mw ≈ 38.4 kDa, and Tg = 34.4 °C. The conjugate was successfully processed into particulate and fibrous coatings on silicone ring substrates using optimized electrohydrodynamic parameters. Morphological analysis by SEM confirmed uniform coating formation, with particulate layers forming porous microsphere structures and fibrous coatings producing entangled nanofiber meshes. In vitro degradation over 56 days resulted in a ~81–94% reduction in Mw and a ~86–93% reduction in Mn, accompanied by distinct morphological transitions, including microsphere coalescence and fiber fusion. Acyclovir release kinetics were morphology-dependent: particulate coatings (R1, R2) released 42.7% and 37.6% of total released drug by day 3, while fibrous coatings (R3, R4) released only 11.9% and 18.0% during the same period. All formulations demonstrated sustained, degradation-driven release through day 56. These findings confirm that both degradation rate and coating architecture govern ACV release, and that electrohydrodynamic methods enable fabrication of covalently loaded coatings with tunable and predictable release profiles for potential long-acting antiviral surface functionalization.

## Figures and Tables

**Table 1:** Electrohydrodynamic processing parameters used for the fabrication of ACV–PLGA coatings and the corresponding morphologies (electrosprayed particles, beaded fibers, or uniform fibers) observed by SEM. The table summarizes how polymer concentration, solvent composition, and applied voltage influence the transition between particulate and fibrous architectures.

Sample	ACV-PLGA Conc. (*w*/*v*%)	Solvent Mixture and Volume Ratio	Employed Voltages (kV)	Morphological Result
ES1	25%	DMF:THF 5:5	9, 11, 13, 15, 17	electrospraying
ES2		DMF:THF 3:5	8, 10, 12, 14, 16	beading
ES3		DMF:CHCl_3_ 5:5	8, 10, 12, 14, 16	electrospraying
ES4		DMF:CHCl_3_ 3:5	7, 9, 11	electrospraying/beading
			13, 15	beading
ES5	30%	DMF:THF 5:5	7, 9, 11, 13, 15, 17	beading
ES6		DMF:THF 3:5	7, 8, 9, 10, 11, 13, 15, 17, 19	beading
ES7		DMF:CHCl_3_ 5:5	9, 11, 13, 15	electrospraying/beading
			17, 19	beading
ES8		DMF:CHCl_3_ 3:5	8, 10, 12, 14, 16, 18	beading
			20	electrospinning
ES9	35%	DMF:THF 5:5	7, 8, 9, 11, 13, 15, 17	beading
ES10		DMF:THF 3:5	8, 9, 10, 11, 12, 13, 14, 15, 16, 17	beading
			18, 19	electrospinning
ES11		DMF:CHCl_3_ 5:5	8, 9, 10, 11, 12, 13	beading
ES12		DMF:CHCl_3_ 3:5	7, 8, 9, 10, 11, 12, 13, 14, 15, 16, 17	beading
			18	electrospinning
ES13	40%	DMF:THF 5:5	7, 9, 11, 13, 15, 17	beading
ES14		DMF:THF 3:5	7, 9, 11, 13, 15, 17	electrospinning
ES15		DMF:CHCl_3_ 5:5	8, 10, 12, 14, 16, 18	beading
ES16		DMF:CHCl_3_ 3:5	8, 10, 12, 14, 16, 18	electrospinning

## Data Availability

The original contributions presented in this study are included in the article/Supplementary Material. Further inquiries can be directed to the corresponding author.

## Supplementary Materials

The following supporting information can be downloaded at: https://www.mdpi.com/article/10.3390/ijms262210983/s1.

## Abbreviations

The following abbreviations are used in this manuscript:

ACV	Acyclovir
PLGA	Poly (lactic-co-glycolic acid)
LA	L-lactide
GA	Glycolide
ROP	Ring-Opening Polymerization
FTIR	Fourier-Transform Infrared Spectroscopy
^1^H NMR	Proton Nuclear Magnetic Resonance
GPC	Gel Permeation Chromatography
DSC	Differential Scanning Calorimetry
SEM	Scanning Electron Microscopy
PDI	Polydispersity Index
Mn	Number-Average Molecular Weight
Mw	Weight-Average Molecular Weight
Tg	Glass Transition Temperature
DMF	Dimethylformamide
THF	Tetrahydrofuran
CHCl_3_	Chloroform
DMSO-d_6_	Deuterated Dimethyl Sulfoxide
PBS	Phosphate-Buffered Saline
HIV	Human Immunodeficiency Virus
HSV	Herpes Simplex Virus
STI	Sexually Transmitted Infection
Sn(Oct)_2_	Tin (II) 2-ethylhexanoate
UV-Vis	Ultraviolet-Visible Spectroscopy
HPLC	High-Performance Liquid Chromatography
ATR	Attenuated Total Reflectance
PCL	Poly (ε-caprolactone)
