# Supplementary material for: Electrohydrodynamic Coating with Acyclovir PLGA Conjugate for Antiviral Functionalization of Medical Surfaces
Source: Int J Mol Sci. 2025 Nov 13;26(22):10983. doi: 10.3390/ijms262210983 (PMC12652063; doi:10.3390/ijms262210983)
Supplement: Supplementary file 1 [file ijms-26-10983-s001.zip › ijms-3905639-supplementary.pdf]

Supplementary Materials for Article

Electrohydrodynamic coating with acyclovir PLGA conjugate for antiviral functionalization of medical surfaces

Tomasz Urbaniak [1], Witold Musiał [2*]

[1] Department of Physical Chemistry and Biophysics, Pharmaceutical Faculty, Wroclaw Medical University, Borowska 211, 50-556 Wrocław, Poland.

* Correspondence: witold.musial@umw.edu.pl

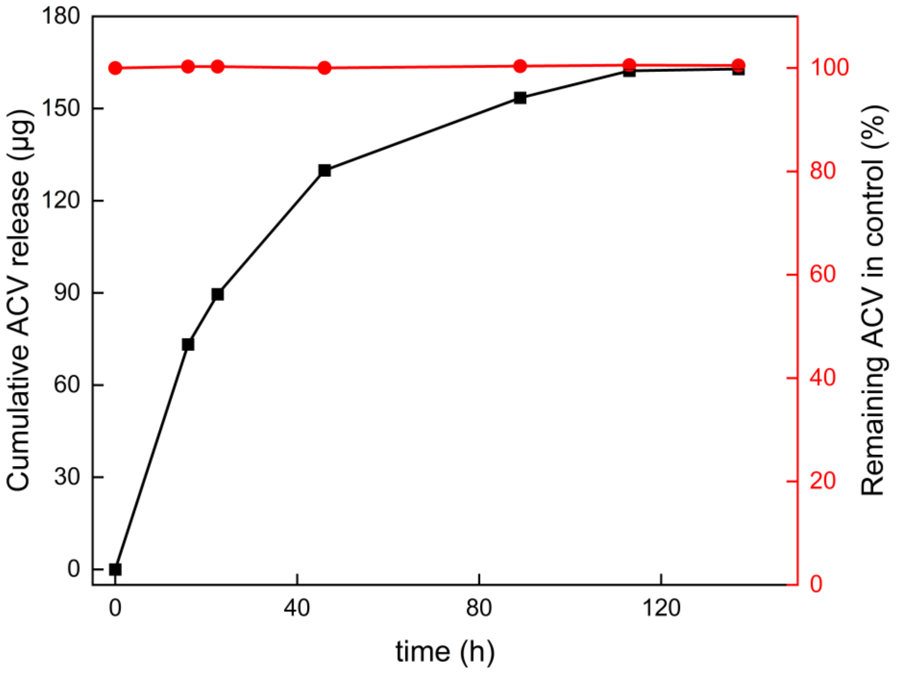

Figure S1. Cumulative ACV release from 100 mg of ACV–PLGA conjugate (black squares, left y-axis) during accelerated degradation in 0.5 M NaOH at room temperature, and stability control of free ACV in same conditions (0.22 mg/mL; red circles, right y-axis).

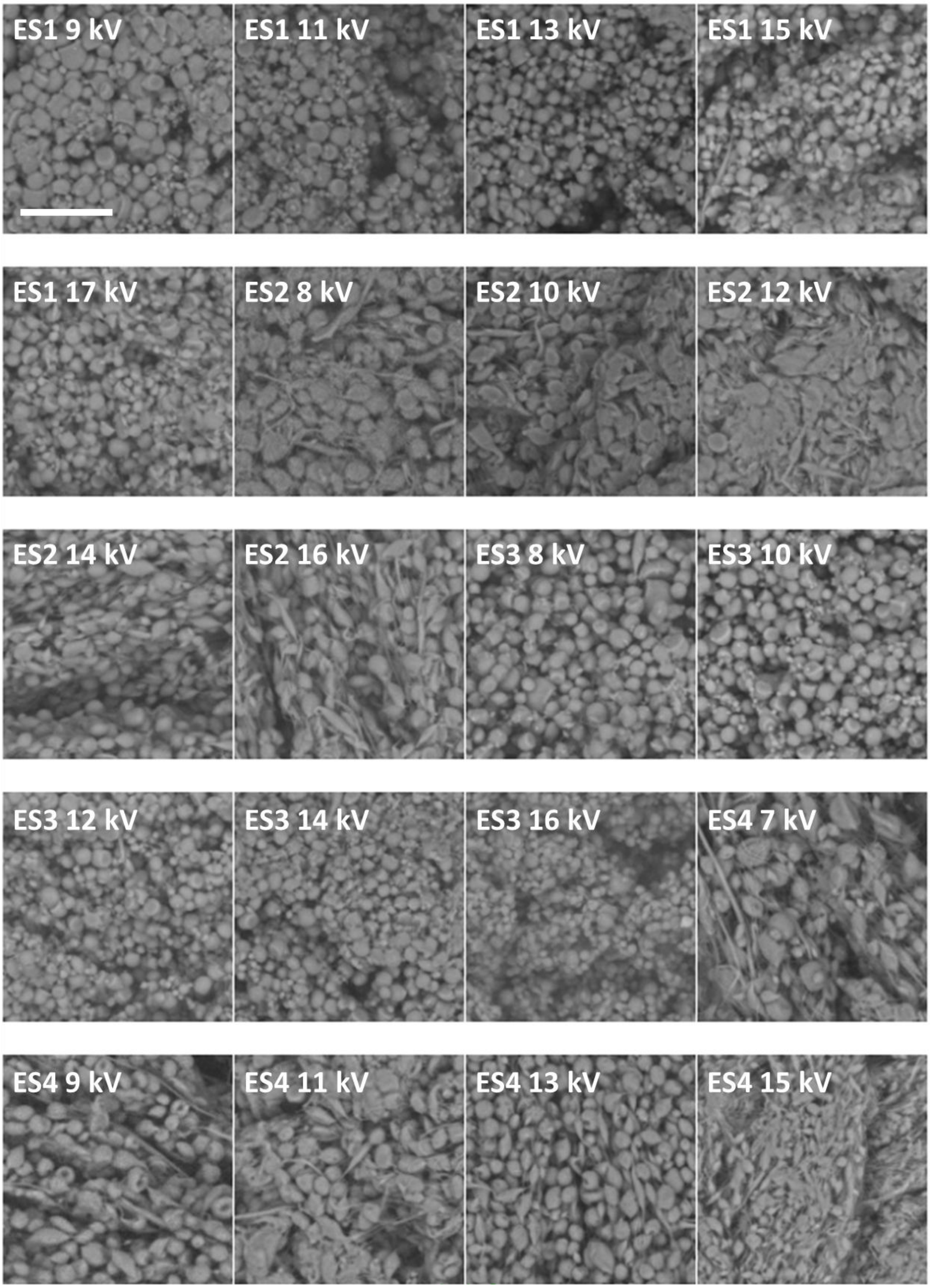

Figure S2. SEM micrographs of electrohydrodynamic processing products obtained from ES1–ES4 solvent systems at different applied voltages. Scale bar: 20 μm.

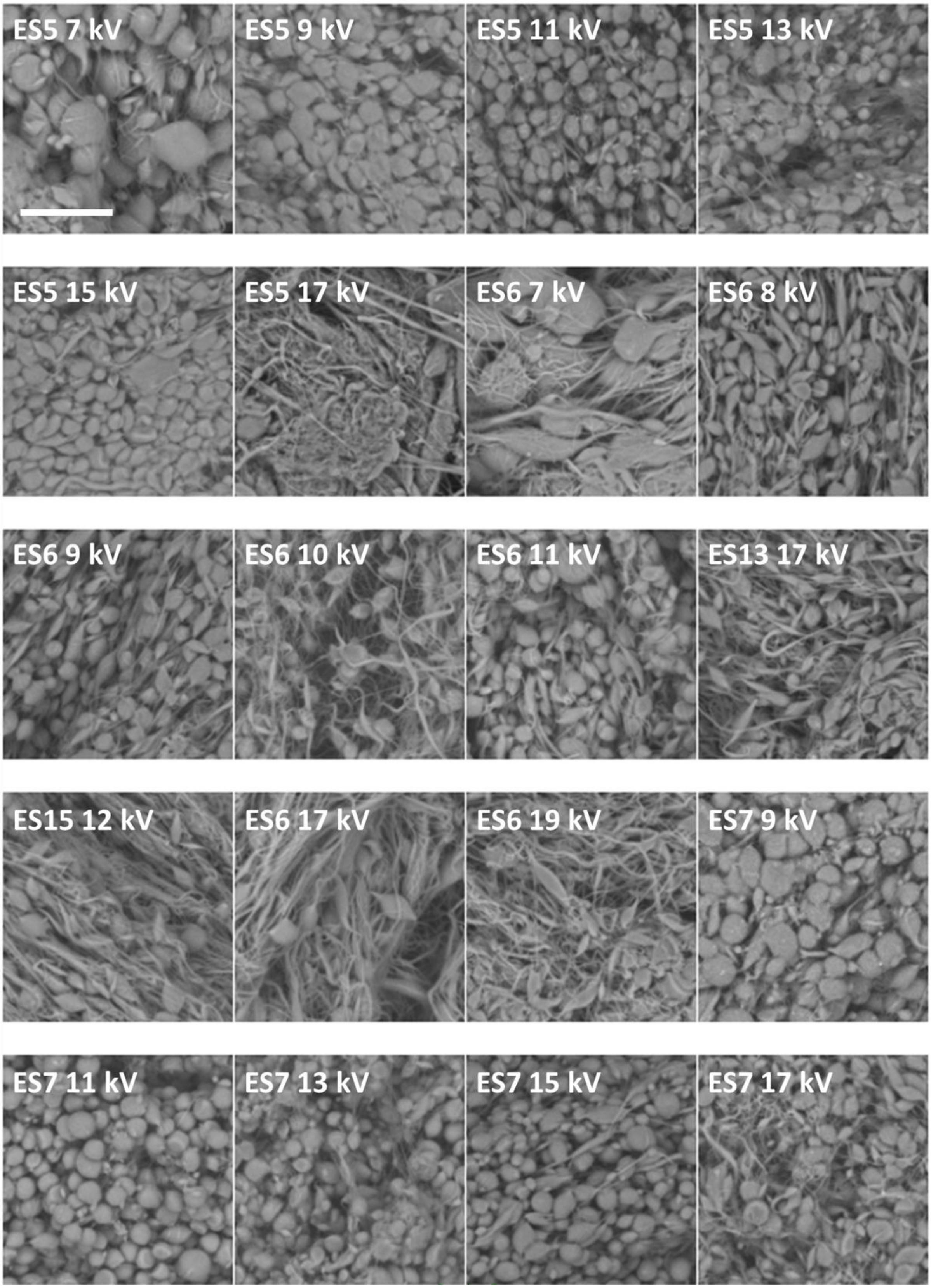

Figure S3. SEM micrographs of electrohydrodynamic processing products obtained from ES5–ES7 solvent systems at different applied voltages. Scale bar: 20 µm.

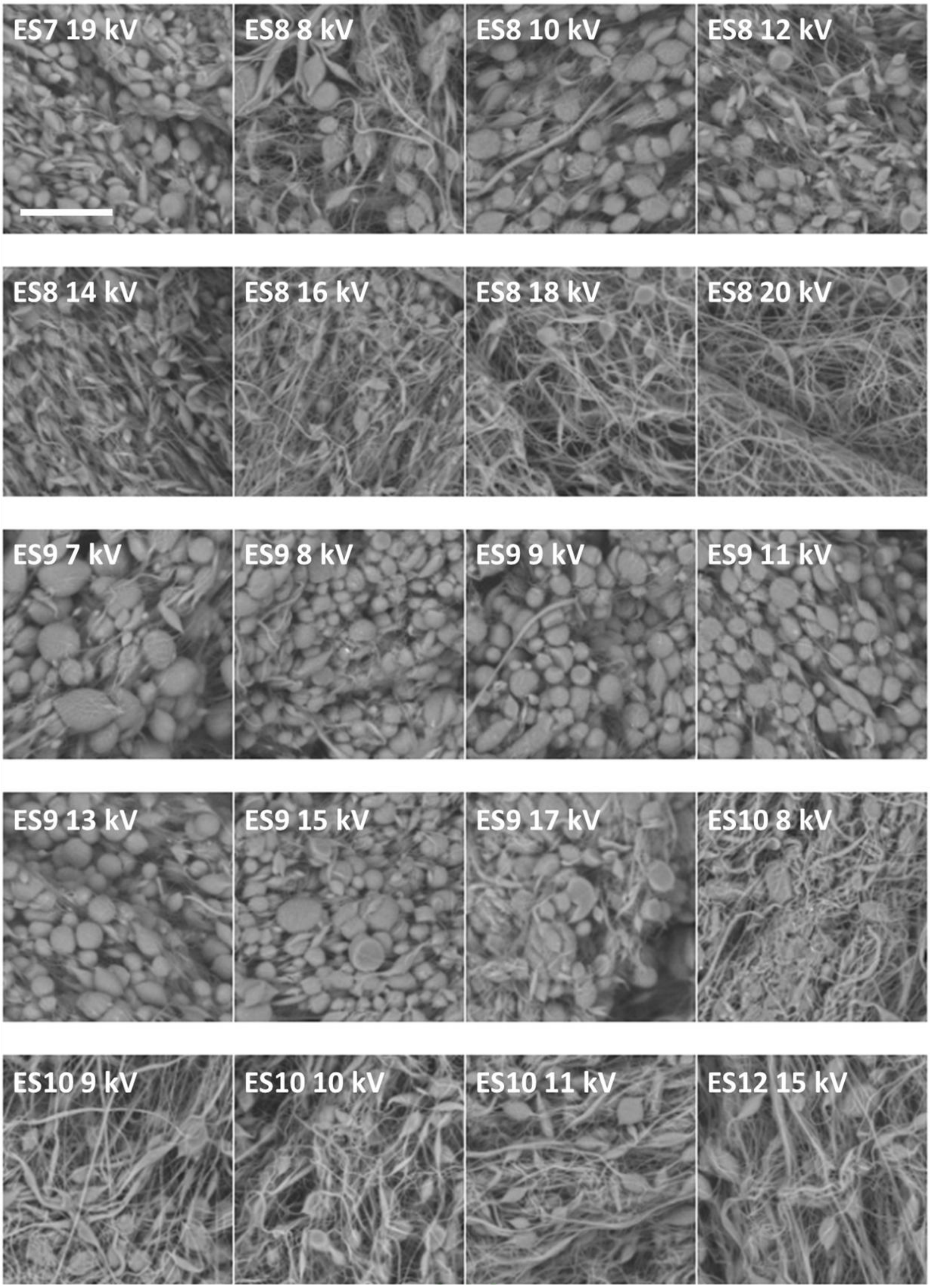

Figure S4. SEM micrographs of electrohydrodynamic processing products obtained from ES7–ES12 solvent systems at different applied voltages. Scale bar: 20 μm.

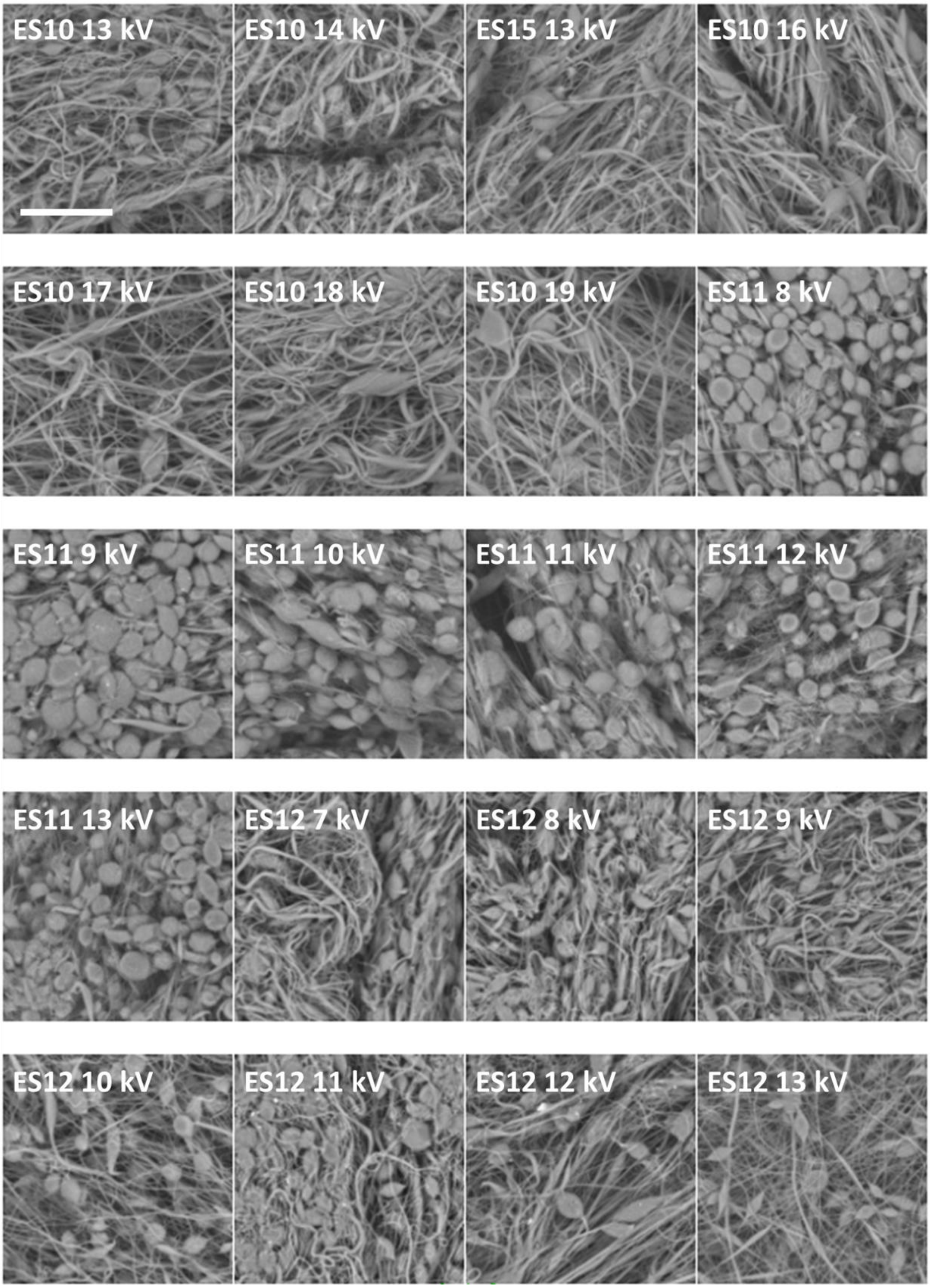

Figure S5. SEM micrographs of electrohydrodynamic processing products obtained from ES10–ES12 solvent systems at different applied voltages. Scale bar: 20 μm.

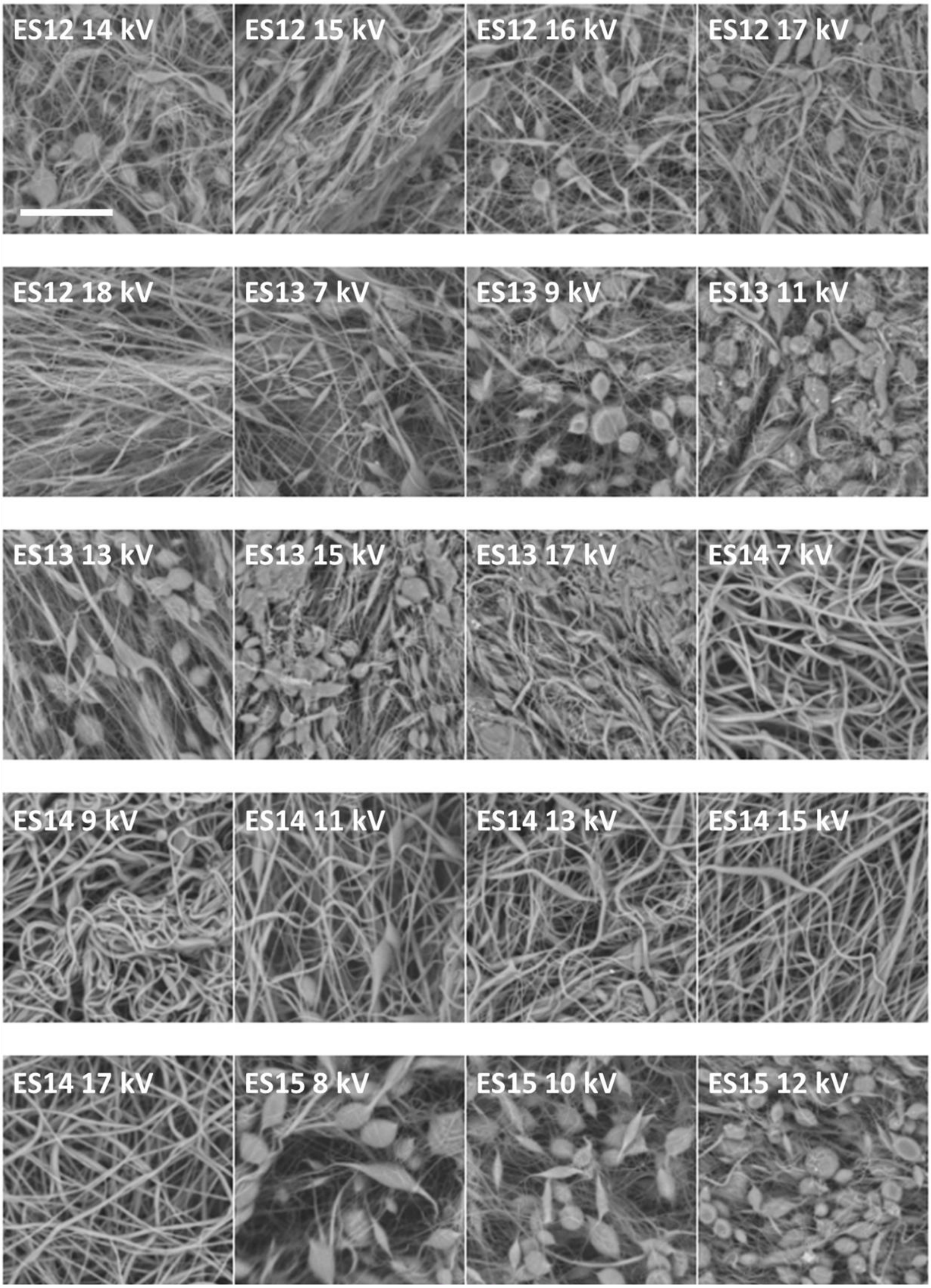

Figure S6. SEM micrographs of electrohydrodynamic processing products obtained from ES12–ES15 solvent systems at different applied voltages. Scale bar: 20 μm.

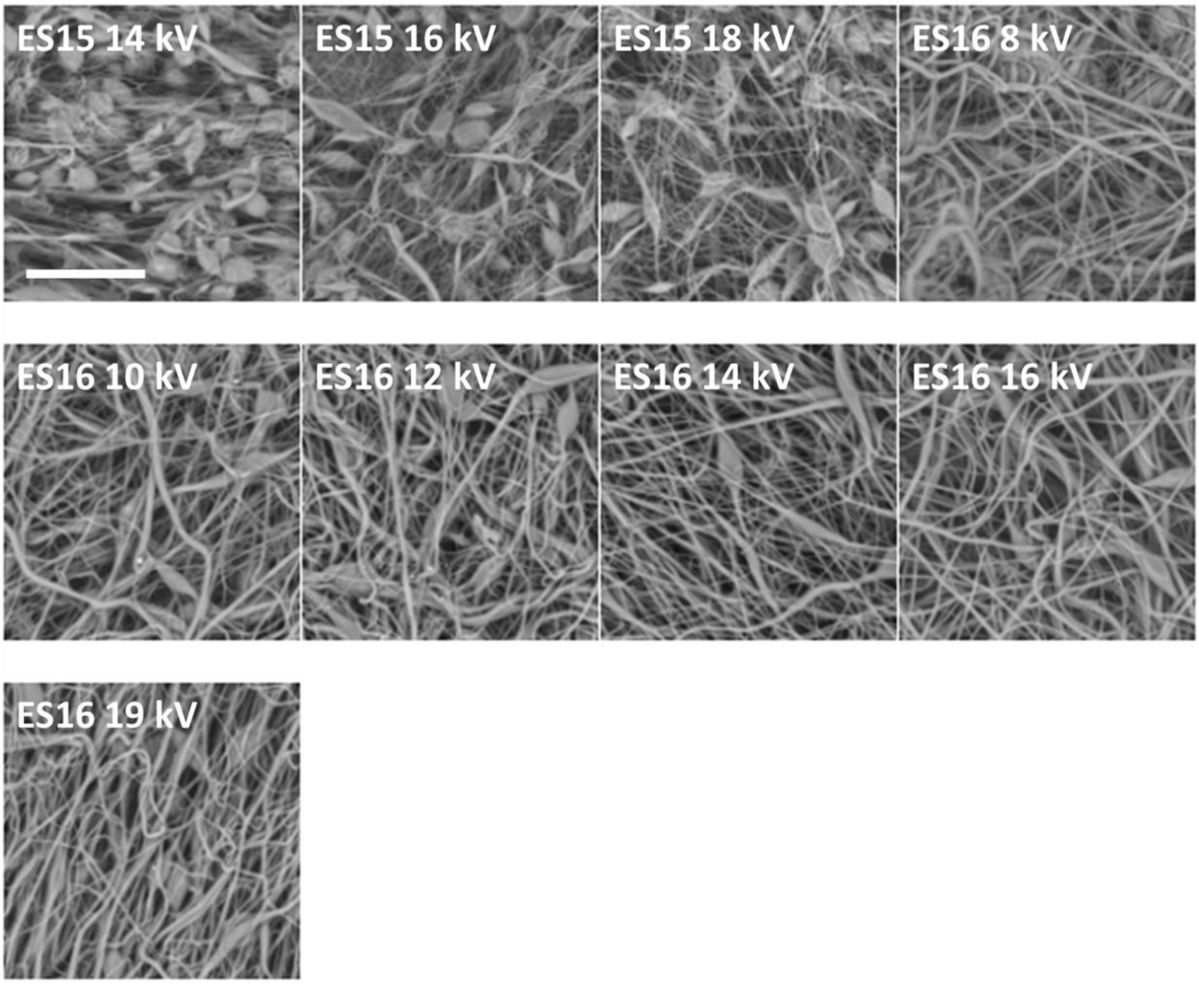

Figure S7. SEM micrographs of electrohydrodynamic processing products obtained from ES15–ES16 solvent systems at different applied voltages. Scale bar: 20 μm.

Table S1. Kinetic parameters (rate constants K or diffusion exponent n and determination coefficients R^2) obtained from linearized zero-order, first-order, Higuchi, and Korsmeyer–Peppas models describing ACV release profiles from conjugate coatings (R1–R4). All data were fitted using fractional release (M_t/M_∞) and free-intercept linear regression.

	**Zero-order** $\frac{M_t}{M_\infty} = K_0 t + b$		**First-order** $\ln\left(1 - \frac{M_t}{M_\infty}\right) = -K_1 t + b$		**Higuchi** $\frac{M_t}{M_\infty} = K_H t^{\frac{1}{2}} + b$		**Korsmeyer–Peppas** $\log\left(\frac{M_t}{M_\infty}\right) = n \log t + \log K$	
	K_0 (day^{-1})	R^2	K_1 (day^{-1})	R^2	K_H (day$^{1/2}$)	R^2	n	R^2
R1	0.0101	0.9332	-0.0245	0.9119	0.0720	0.9273	not applicable	
R2	0.0116	0.9640	-0.0424	0.9485	0.1105	0.9806	not applicable	
R3	0.0115	0.9447	-0.0247	0.8917	0.1085	0.9397	0.5668	0.9733
R4	0.0100	0.9242	-0.0203	0.8552	0.0908	0.8584	0.3533	0.9142
